# Marked sex differences are observed in heroin acquisition and affective states in rats but converge to similar levels of footshock stress-induced reinstatement

**DOI:** 10.1007/s00213-025-06920-w

**Published:** 2025-11-03

**Authors:** Claire Deckers, Zeinab Ahmed, Chen Li, Lynn G. Kirby

**Affiliations:** 1 Center for Substance Abuse Research, Lewis Katz School of Medicine at Temple University, Philadelphia, USA

**Keywords:** Heroin use disorders, Sex differences, Ultrasonic vocalizations, Stress

## Abstract

**Rationale:**

Negative affective states, particularly those driven by stress, are known to trigger the development of opioid addiction and relapse. Previous preclinical research has identified sex differences in vulnerability to stress and resulting compulsion to consume drugs.

**Objectives:**

To investigate sex differences in heroin self-administration, stress-induced reinstatement, and related affective state in rats.

**Methods:**

22- and 50-kHz ultrasonic vocalizations (USVs), reflecting negative and positive affective state, respectively, were recorded during a heroin self-administration and footshock stress-induced reinstatement model in Sprague-Dawley rats. We hypothesized that females would display increased vulnerability towards heroin use disorder-like profiles.

**Results:**

Females showed elevated acquisition of heroin self-administration, but behavioral patterns were not linked to gonadal hormone levels. During heroin acquisition, males emitted significantly more 50 kHz calls compared to females, suggesting positive reinforcement-driven intake. We observed successful stress-induced reinstatement in both sexes, with males and females reaching similar overall numbers of active lever presses by the end of the reinstatement session. However, they appear to do so via different strategies, with females displaying “frontloading”, rapidly lever pressing in the first 20 min of the behavioral session, perhaps indicating differential stress vulnerability or differences in underlying neural encoding of stressor/reward value. Successful stress-induced reinstatement in both sexes is accompanied by a distinct pattern of USVs, whereby a high number of anticipatory 50 kHz calls are emitted prior to the onset of reinstatement sessions, followed by 22 kHz call production by males during reinstatement sessions, perhaps reflecting drug seeking to relieve negative affect or negative affective responses to drug unavailability.

**Conclusions:**

These findings indicate sex-specific vulnerabilities to development of heroin use disorders.

## Introduction

Heroin use disorders (HUDs) are severe psychiatric conditions marked by compulsive heroin use, tolerance, and dependence (Strang et al., 2020). Although treatment protocols exist, including replacement therapy and behavioral support, relapse rates remain high, with 60–75% of individuals relapsing within a year of treatment (Maehira et al., 2013; Kadam et al., 2017; Nunes et al., 2018). Negative reinforcement is proposed to play a key role in both active drug consumption and relapse, whereby drug use functions as a form of “self-medication” to provide relief from negative physiological and psychological states, particularly those associated with stress (Solomon, 1980; Shaham et al., 2000; Mantsch et al., 2016). There is considerable preclinical and clinical evidence to support this association between stress, heroin usage, and relapse (Yu et al., 2007; Sinha, 2008; Preston & Epstein, 2011; Mantsch et al., 2016; Moran et al., 2018; Wemm & Sinha, 2019; Stafford et al., 2019; Mantsch, 2022). Understanding the mechanisms of stress-induced relapse could lead to better pharmacotherapies for treatment.

This issue is particularly salient given that drug use in men and women tends to be driven by different motivational factors. In clinical populations, women largely report negative reinforcement driven-drug use and relapse and largely cite stress as a preeminent driver in these behaviors (Yu et al., 2007; Becker & Hu, 2008; Back et al., 2011; Kennedy et al., 2013; Kadam et al., 2017; Nunes et al., 2018; Moran et al., 2018; McHugh et al., 2018, 2021; Becker & Chartoff, 2019; Fonseca et al., 2021; Sullivan, 2022). Conversely, men are more likely to report drug use related to positive reinforcement (i.e. hedonic experience of drug consumption, social benefits, propensity towards risk-taking, etc.) (McHugh et al., 2018; Becker & Chartoff, 2019). Furthermore, numerous clinical and preclinical studies have noted sex-specific vulnerability to the development of HUDs, with females being overall more vulnerable relative to males according to a variety of criteria, including more rapid acquisition of heroin self-administration and progression to “addiction-like” stages (Becker & Hu, 2008; Becker & Chartoff, 2019; Knouse & Briand, 2021).

In this study, we seek to outline baseline sex differences in heroin consumption and stress-induced reinstatement behaviors, as well as affective states that contribute to these behaviors. We employed a translationally relevant long-intermittent access self-administration schedule which induces binge responding in animals, modeling patterns of human consumption (Blackwood et al., 2020; Fragale et al., 2021; D’Ottavio et al., 2023). A microanalysis of self-administration sessions was conducted to assess sex differences in voluntary heroin consumption patterns. To explore reinstatement behaviors, we utilized a footshock stress-induced reinstatement model, accompanied by behavioral microanalysis, to analyze sex-specific seeking behaviors after stress exposure. Though other studies have examined sex differences in footshock-stress induced reinstatement following short access to oral oxycodone, no studies have explored sex differences in footshock stress-induced reinstatement of heroin seeking, highlighting a significant gap in the field (Fulenwider et al., 2020). Accompanying these behavioral assays, 50- and 22-kHz ultrasonic vocalizations, reflective of positive and negative affective states (Wöhr & Schwarting, 2013; Lenell et al., 2021; Premoli et al., 2023), respectively, were recorded during self-administration and footshock stress-induced reinstatement in order to compare sex differences in affective states related to heroin consumption and reinstatement.

We hypothesized that females would show increased baseline heroin consumption and heightened stress-induced seeking of heroin, accompanied by stronger negative affective responses to stress relative to males. This work expands upon previous literature by defining sex differences in heroin-related behaviors and affective states across drug use cycles using a model with improved translational validity.

## Methods

### Subjects

Male and female Sprague Dawley rats (7–8 weeks old, 200–350 g; Taconic Biosciences, Germantown, NY) were housed on a reverse 12 h light/dark cycle (lights on at 21:00, lights off at 09:00) in standard housing conditions with ad libitum access to food and water. Subjects were group housed by sex with 2 rats per cage prior to surgeries for intravenous catheterization. A total of 36 rats were used for this study (15 M, 21 F). All behavioral testing was conducted during the rat dark cycle. All procedures were conducted in accordance with policies set forth by Temple University Institutional Animal Care and Use Committee and the National Research Council’s *Guide for the Care and Use of Laboratory Animals*.

### Intravenous catheterization surgeries

All subjects were given at least 1 week to acclimate prior to surgeries. Following sufficient acclimatization time, rats were anesthetized and a polyurethane i.v. catheter was implanted into the right jugular vein. Surgical protocols, patency confirmation, and maintenance followed standard procedures (Li & Frantz, 2009; Li & Kirby, 2016; Li et al., 2025). All subjects received subcutaneous doses of ketoprofen (5 mg/kg) prior to surgery and on postoperative day 1. Subjects were given one week of recovery following surgery prior to initiation of behavioral experiments.

### Drugs

Heroin HCl (generously supplied by the National Institute of Drug Abuse Drug Supply Program) was dissolved in saline (0.2 mg/ml) and infused in a volume of 0.1 mg/kg/infusion i.v. for the first week of self-administration, and a volume of 0.05 mg/kg/infusion i.v. for subsequent behavioral testing. Doses were chosen based upon their efficacy in this model as described previously (Li et al., 2025).

### Heroin self-administration, extinction, and stress-induced reinstatement paradigms

**Fig. 1:**
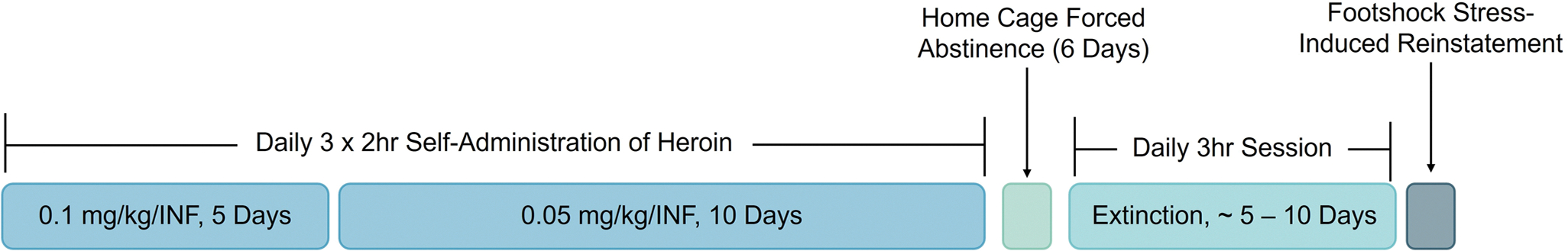
Timeline of Study Progression. Rats were trained to self-administer heroin with daily 3 × 2 h sessions at an initial dose of 0.1 mg/kg/INF for 5 days and maintained at 0.05 mg/kg/INF for 10 days. Extinction was conducted until rats met extinction criteria (> 80% decrease in lever pressing from baseline for 3 days in a row). Footshock stress-induced reinstatement was conducted the following day, consisting of 15 min of random, intermittent footshock at 0.5 mA, followed by a 10 min break. Reinstatement sessions were then initiated under extinction conditions. INF = infusion

Behavioral timelines can be found in Fig. 1; additional information surrounding self-administration paradigms can be found in the Supplemental Methods. Rats were given a week to recover post-surgery prior to beginning behavioral paradigms. Following this recovery period, rats were trained to self-administer heroin in a long intermittent-access paradigm, consisting of 3 × 2 h behavioral sessions, separated by 30 min (Blackwood et al., 2020; Li et al., 2025). During 30-minute breaks, all levers were retracted and house lights were turned off. Behavioral sessions took place in 5-day blocks, with 2 days off in-between. For the first five days of acquisition, lever pressing on the active lever was reinforced by i.v. infusion of heroin (0.1 mg/kg/infusion) under a fixed-ratio 1 (FR1) schedule of reinforcement. Long access FR1 behavioral sessions continued for an additional 10 sessions on a lower dose of heroin (0.05 mg/kg/infusion). Successful acquisition of heroin self-administration was considered to be an average of 10 infusions per individual session, and more than 70% of responding on the active lever versus inactive lever to demonstrate successful lever discrimination; these criteria are similar to those used in other self-administration studies for drugs of various classes (Lynch & Carroll, 1999; David et al., 2001; Maccioni et al., 2008; Contet et al., 2010). If subjects did not reach these criteria, they were removed from the study (6 of 36 rats removed due to failure to discriminate active vs. inactive levers).

Following acquisition sessions, subjects underwent 6 days of home-cage forced abstinence, before beginning extinction paradigms (Shalev et al., 2001). During extinction periods, all operant chamber conditions remained consistent, but active lever presses did not result in any cue or reinforcer presentation. Extinction sessions were 3 h long, and were again run in 5-day blocks, with two days off in-between. Subjects continued in the extinction conditions until extinction criteria were met, defined a priori as a > 80% reduction in active lever pressing from the first day of extinction for 3 consecutive sessions (Pockros-Burgess et al., 2014).

Following successful extinction of heroin seeking, stress-induced reinstatement paradigms took place. Subjects were placed in the operant chamber for a 10-minute acclimation period, after which house lights were turned on and they were exposed to 15 min of random, intermittent footshock (0.5 mA; 0.5 s on; mean off period of 40 s; range of 10–70 s). Following footshock, subjects remained in the operant chamber for an additional 10 min, to allow for recording of anticipatory USVs, during which time all house lights were turned off and levers retracted. 3-hour reinstatement behavioral sessions then began, indicated by house lights turning on and levers extending. During reinstatement behavioral sessions, subjects could lever press freely under extinction conditions (i.e. lever pressing produces no cue or reinforcer presentation). Levels of reinstatement were assessed by examination of relative increases in active lever pressing during reinstatement behavioral sessions as compared to the final day of extinction (Shaham & Stewart, 1995; Shaham et al., 2000; Shalev et al., 2001; Mantsch et al., 2016).

### Ultrasonic vocalization measurement

USVs were recorded as subjects sat in operant chambers for 10 min prior to the start of behavioral sessions, in order to measure anticipatory USVs. USVs were also recorded for the first 20 min of each behavioral session to examine drug/cue evoked USV emission. On reinstatement days, USVs were recorded in the 10-minute interim period following footshock to capture anticipatory USVs, as well as for the first 20 min of reinstatement behavioral sessions. In a small subset of subjects, additional recording timepoints were added, where USVs were measured for 10 min prior to the beginning of footshock and were measured continuously through the 15-minute footshock sessions as well (see Supplemental Information).

### Estrous cycle measurement

Estrous cycles were tracked in all female subjects via assessment of vaginal cytology. Collection of vaginal cells followed standard vaginal lavage procedures (Robert et al., 2021). Additional details can be found in the Supplemental Methods section.

### Data analysis

All data analysis was conducted in GraphPad Prism (Dotmatics, San Diego, CA, v.10.4.1). For all data, outlier analysis was run using built-in ROUT analysis. Normality was then assessed using both D’Agostino-Pearson omnibus and Shapiro-Wilk tests to ensure proper statistical test selection. Specific statistical tests are noted in the results section associated with each set of data, but generally, behavioral data were analyzed via two- or three-way (factors of lever type, sex, and time) repeated measures (RM) ANOVA or comparable non-parametric tests, with Sidak’s or Tukey’s multiple comparisons conducted *post-hoc* if appropriate. USV data were analyzed using three-way RM ANOVA (factors of sex, call type, and time) for calls during acquisition, and via two-way RM ANOVA for calls during reinstatement. Correlations between various behavioral and USV profiles were examined using simple linear regression; data are reported as Pearson’s correlations. No corrections were performed for the correlations.

### Behavioral microanalysis: cumulative record and onset skew

Behavioral microanalysis was performed at two points: Day 11 of self-administration to examine active lever pressing and infusion patterns and the reinstatement day (Day 35–49) to examine active lever pressing patterns during reinstatement behavioral sessions. Parameters of interest (i.e. infusion or active lever press, along with time of event) was extracted from SoftCR (v 4.20.0.0, MedAssociates, Inc.). Average curves for each sex were generated in GraphPadPrism (Dotmatics, San Diego, CA, v.10.4.1). To examine active lever pressing patterns during acquisition, active lever presses/time was graphed using GraphPadPrism. Active lever pressing exhibited a non-linear pattern, characterized by elevated responding at the beginning of each session (i.e., frontloading). To quantify frontloading behavior during acquisition, we calculated an onset skew index, defined as the proportion of total active lever presses occurring within the first five minutes of each session (D’Ottavio et al., 2023; McNamara et al., 2025). For acquisition data, sex and session differences in onset skew were analyzed using a two-way RM ANOVA. To examine infusion patterns during acquisition, number of heroin infusions/time was graphed using GraphPadPrism. Upon visualization, infusion data for Day 11 of self-administration was linear in nature; therefore, we performed simple linear regression for infusion curves during each of the three daily sessions and used built-in ANCOVA analysis in Prism to assess if slopes for the curves were significantly different from each other.

For active lever pressing patterns during reinstatement, active lever presses/time was graphed using GraphPad Prism. Onset skew analysis was then performed, as described above. Sex differences in onset skew were assessed using an unpaired student’s t test. We then examined time to meet reinstatement criteria, defined by two components: (1) a minimum of 10 active lever presses during the reinstatement session, and (2) active lever pressing that reached at least 150% of baseline levels (i.e., a 50% increase relative to baseline). As reinstatement criteria within opioid literature have not been well-established, these criteria were adapted from psychostimulant literature to better fit heroin consumption patterns (Soria et al., 2008; Havlickova et al., 2018). Time to meet reinstatement criteria was then graphed as a survival curve; differences in survival curves were then assessed using Chi-square tests in GraphPad Prism.

### Ultrasonic vocalization analysis

All USV files were imported from RavenPro to DeepSqueak (v3.1.0) in .wav format. USVs were analyzed using modified versions of preset neural networks (“Rat Detector YOLO R1.mat” to analyze 50 kHz calls and “Long Rat Detector YOLO R1.mat” to analyze 22 kHz calls) that have been trained via supervised deep learning methods to identify 50 kHz and 22 kHz USVs, and further to distinguish these calls from background noise. Identification of all calls was checked manually to confirm that all USVs were detected, and further to ensure that false positives were not included (Coffey et al., 2019). Parameters extracted for analysis included number of anticipatory USVs emitted, and number of USVs emitted during behavioral sessions. USV profiles were also examined in correlation with various behavioral phenotypes (see details of analysis below).

To examine USVs in the context of drug infusions, the number of USVs 5 s before, during, and 5 s after infusions (detected by microphones as noise from the infusion pump) was recorded for each subject. Non-specific calling was considered to be any USVs occurring outside of these contexts. Call rates were then normalized to allow for direct comparison of calling during these time points (see Supplemental Methods for details).

## Results

### Heroin self-administration

**Fig. 2:**
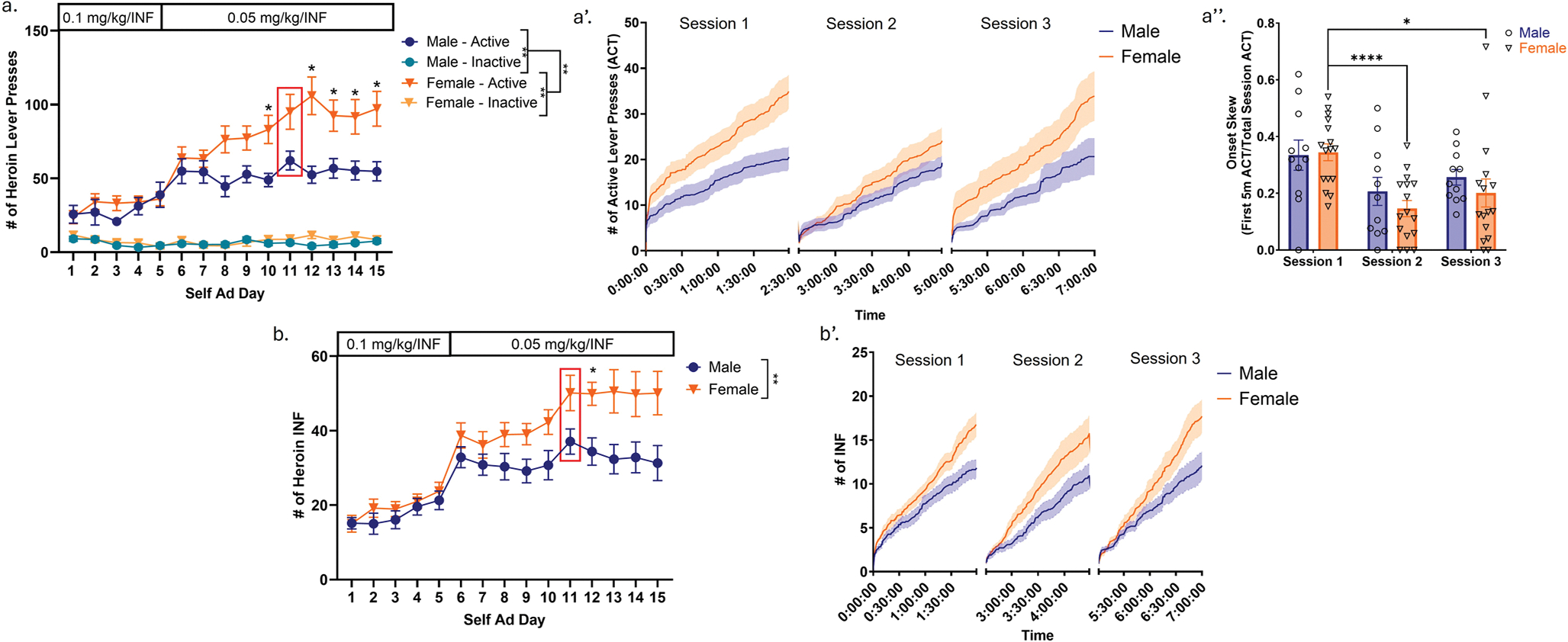
Females show significantly higher intake and escalation of heroin-self administration compared to males. **a**. Lever pressing in males and females during acquisition. Boxed area indicates selected day of analysis shown within a’. a’. Active lever pressing patterns during individual long-intermittent access sessions during Monday of Week 3 (Day 11). a”. Onset skew analysis to quantify active lever frontloading patterns observed in b’. Quantified as active lever presses in first five minutes of session as a proportion of total session active lever presses. Females showed significantly greater onset skew of active lever presses during Session 1 compared to Session 2 and 3. **b.** Heroin infusions in males and females during acquisition. Boxed area indicates selected day of analysis shown within b’. b’. Heroin infusion patterns during individual long-intermittent access sessions during Monday of Week 3 (Day 11). Both males and females consume heroin steadily throughout all daily sessions. We performed linear regression analyses for all sessions and compared slopes to assess differences in rates of heroin consumption. Females consumed heroin at a significantly higher rate than males for all three sessions. Consumption patterns did not significantly differ within sexes between sessions. See text for statistical analysis and details. Error bars represent SEMs. * indicates p < 0.05 assessed by comparison of females vs. males by post-hoc Sidak’s (Panel a) or Tukey’s (Panel b and b”) multiple comparisons test. ** indicates main effects in two- or three-way ANOVA (p < 0.01). **** indicates p < 0.0001 by comparison of sessions by post-hoc Tukey’s (Panel b”) multiple comparisons test. INF = infusion. ACT = active lever press

During acquisition, we observed that both active lever presses (ACT) and, consequently, heroin infusions (INF), significantly increased over the course of the three-week period (Three-way RM ANOVA; ACT: F(14, 406) = 17.42, *p* < 0.0001; Two-way RM ANOVA; INF: F(4, 93) = 26.99, *p* < 0.0001) (Fig. 2a & b). Additionally, we also observed a significant main effect of sex on both parameters (Three-way RM ANOVA; ACT: F(1, 288) = 9.215, *p* = 0.0026; Two-way RM ANOVA; INF: F (1, 29) = 6.674, *p* = 0.0151), whereby females performed significantly more active lever presses and consumed more heroin relative to males (Fig. 2a & b). Further analyses revealed a significant interaction between time and sex for both parameters (Three-way RM ANOVA; ACT: F(1, 288) = 7.097, *p* = 0.0082; Two-way RM ANOVA; INF: F(14, 377) = 1.922, *p* = 0.0231), reflecting a sex-related divergence in heroin seeking and consummatory patterns that emerged over the course of Weeks 2 and 3 of self-administration. Due to the observation of a significant interaction effect in both parameters, we performed post-hoc multiple comparisons analysis (Tukey’s multiple comparisons used for ACT; Sidak’s multiple comparisons used for INF), indicating that females showed significantly elevated active lever presses relative to males on Days 10, 12, 13, 14, and 15 (Fig. 2a), as well as significantly elevated heroin consumption relative to males on Day 12 of acquisition (Fig. 2b).

To further explore sex-specific patterns of heroin consumption, microanalysis of active lever pressing and heroin infusion patterns were assessed on Day 11 of self-administration. This time point was chosen as all included subjects had successfully met acquisition requirements by this day. Notably, females demonstrated overall higher rates of lever pressing and heroin consumption, consistent with the observed overall sex differences in these parameters (Fig. 2a’ and b’). This visualization also revealed that both males and females exhibited “frontloading” of active lever pressing, particularly during Session 1 (Fig. 2a’). Onset skew analysis (calculated as the proportion of total active lever presses occurring within the first 5 min of each session) was performed to allow for quantification of frontloading behavior. This analysis demonstrated pronounced onset skew during Session 1 (Fig. 2a”). Males completed 0.335 ± 0.053 (Mean ± SEM; expressed as a proportion of total active lever presses) of their active lever presses during this early interval, and females completed 0.345 ± 0.03 of their lever presses in this period. Frontloading behavior was also observed during Sessions 2 and 3, though to a lesser extent in both sexes (Session 2: males = 0.207 ± 0.05, females = 0.146 ± 0.028; Session 3: males = 0.256 ± 0.027, females = 0.201 ± 0.049). Accordingly, analysis demonstrated a significant main effect of session on onset skew (Two-way RM ANOVA; F(2, 48) = 11.10, *p* = 0.0001), but no effects of sex or interactions between the two parameters (Two-way RM ANOVA; Sex: F(1, 25) = 0.5683, *p* = 0.4580; Session x Sex: F(2, 48) = 0.5628, *p* = 0.5665). Post-hoc Tukey’s indicated that significant session effects were primarily driven by females, who showed significantly higher onset skew in Session 1 relative to Sessions 2 and 3 (Tukey’s; Session 1 vs. Session 2: Mean Difference = 0.7188, *p* = 0.0035; Session 1 vs. Session 3: Mean Difference = 0.07188, *p* = 0.0010) (Fig. 2a”). However, this onset skew pattern did not translate to heroin consumption, as intake remained consistent throughout the sessions for both sexes (Fig. 2b’). To further quantify these differences, linear regression analyses of heroin intake patterns were conducted for each session, revealing that the slopes of cumulative infusions significantly differed between males and females (Linear regression followed by ANCOVA; Session 1: ♂ slope = 0.08436, ♀ slope = 0.1191, F(1, 3664) = 134.3, *p* < 0.0001; Session 2: ♂ slope = 0.08935, ♀ slope = 0.1258, F(1, 2908) = 45.61, *p* < 0.0001; Session 3: ♂ slope = 0.08605, ♀ = 0.1396, F(1, 3244) = 128.0, *p* < 0.0001). Infusion rates did not significantly differ across sessions.

Vaginal swabs in females were collected and cytological analysis was conducted to determine estrous cycle phase; impacts of estrous cycle phase were examined in relation to heroin self-administration patterns during Week 3 of self-administration, at which point behavioral patterns were relatively well-established and stable. No significant differences across estrous cycle phases were found in terms of number of active lever presses (One-way ANOVA; F(3, 49) = 1.333, *p* = 0.2746) or number of infusions (One-way ANOVA; F(3, 57) = 0.2806, *p* = 0.8392). Estrous cycle dysregulation was observed in some females; subjects not displaying consistent estrous cyclicity were not included in the above analysis (4 of 17 subjects not included due to inconsistent cycling). We also examined motivation to obtain heroin via progressive ratio (PR) testing in a subset of subjects (n(♂) = 11, n(♀) = 12). No significant sex differences were observed in terms of number of heroin infusions (Twoway RM ANOVA; F(1, 23) = 1.160, *p* = 0.2926), break point (Mann-Whitney U = 10, *p* = 0.6905), or number of active lever presses (Two-way RM ANOVA; F(1, 23) = 0.04618, *p* = 0.8317). No estrous cycle influences on behavior during PR were observed in females in terms of number of heroin infusions (Kruskal-Wallis = 1.515, *p* = 0.6788), break point (Kruskal-Wallis = 2.787, *p* = 0.4257), or number of active lever presses (Kruskal-Wallis = 5.939, *p* = 0.1146).

### USVs during heroin self-administration

**Fig. 3:**
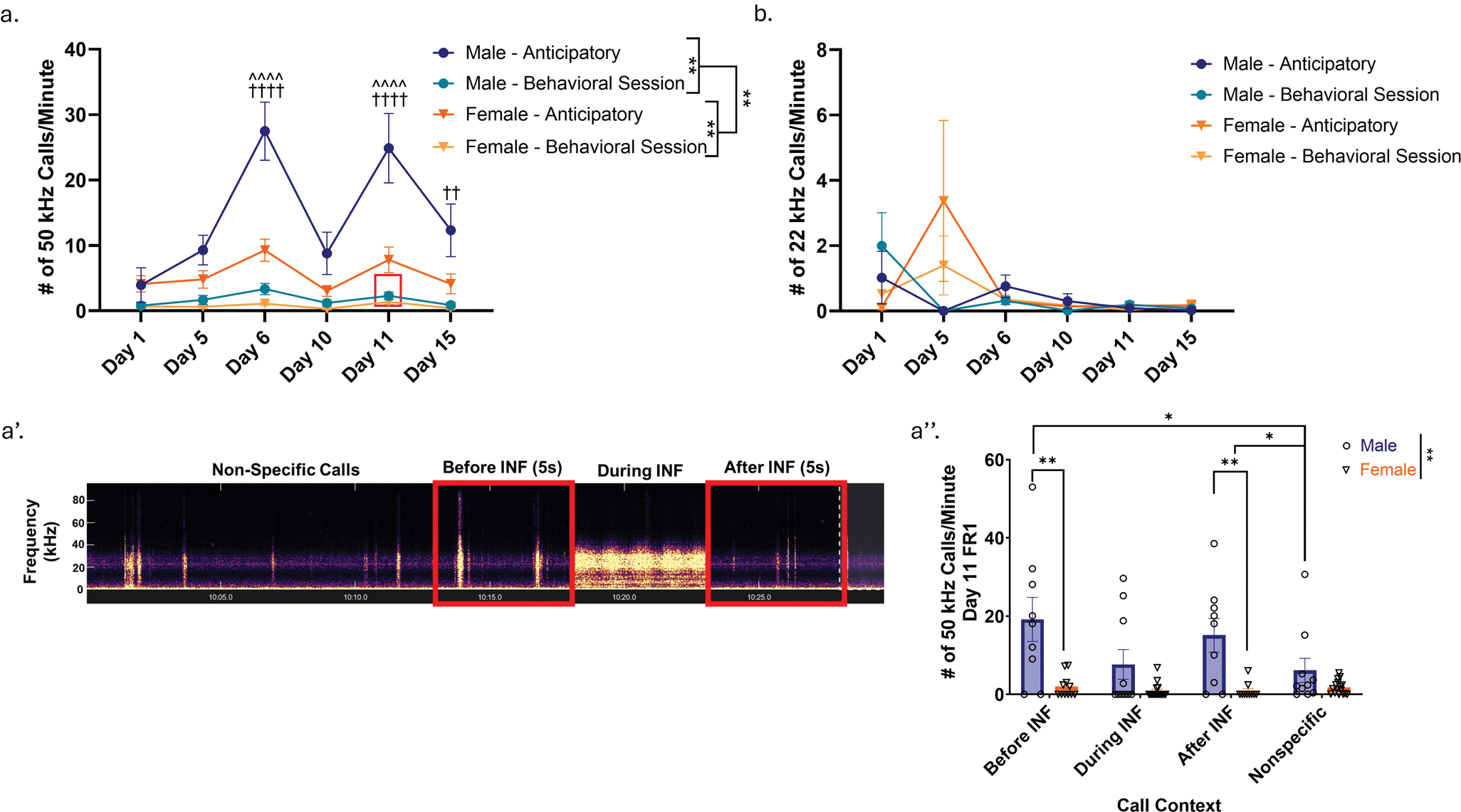
Males emit more “positive” 50 kHz USVs during acquisition of heroin self-administration compared to females, with call emission tied to infusion experience. a. 50 kHz USV emission during acquisition; analyzed on Mondays and Fridays over the course of self-administration. Boxed region indicates day of 50 kHz behavioral session call analysis performed in a’ and a”. n(♂) = 14, n(♀) = 17. a’. Timeline for behavioral session call analysis on Day 11 of acquisition. Call rates were examined before, during, and after heroin infusion, as well as non-specific calling outside of these contexts. Call rates were normalized based upon amount of time to allow for direct comparison (5s for before/after infusion; calculated based upon pump time during infusion; remaining time for non-specific calling derived from the above contexts). Note that while the figure illustrates the temporal structure of the analysis, individual calls are not visible due to the long timescale. a”. 50 kHz USV emission in relation to “infusion context”. Males call at significantly higher rates than females before and after infusions. Additionally, males call at significantly higher rates before and after heroin infusion compared to call rates outside of infusion contexts. n(♂) = 10, n(♀) = 16. b. 22 kHz USV emission during acquisition; analyzed on Mondays and Fridays over the course of self-administration. n(♂) = 14, n(♀) = 17. Error bars represent SEMs. * indicates p < 0.05 by post-hoc Tukey’s Multiple Comparisons. ** indicates main effects in two- or three-way ANOVA or post-hoc Tukey’s multiple comparisons (p < 0.01). †† indicates p < 0.01 by post-hoc Tukey’s (Panel a, Male Anticipatory Calls vs. Male Behavioral Session Calls). †††† indicates p < 0.001 by post-hoc Tukey’s (Panel a, Male Anticipatory Calls vs. Male Behavioral Session Calls). ^^^^ indicates p < 0.001 by post-hoc Tukey’s (Panel a, Male Anticipatory Calls vs. Female Anticipatory Calls). INF = infusion. s = seconds

USV emissions on Mondays and Fridays during self-administration were analyzed, with significantly more anticipatory 50 kHz USVs emitted relative to USVs emitted during the behavioral session in both sexes (Three-way RM ANOVA; F (1, 29) = 70.46, *p* < 0.0001) (Fig. 3a). Additionally, males exhibited significantly more 50 kHz USVs compared to females (Three-way RM ANOVA, main effect of sex; F (1, 93) = 15.20, *p* = 0.0002), an effect that appears to be driven particularly by the high number of anticipatory USVs emitted by males, reflected by a significant interaction between sex and call type (Three-way RM ANOVA; F (1, 93) = 13.13, *p* = 0.0005) (Fig.3a). Furthermore, there was a significant main effect of time (Three-way RM ANOVA; F (5, 145) = 11.04, *p* < 0.0001), as well as a significant interaction between Time x Sex (Three-way RM ANOVA; F (5, 93) = 4.099, *p* = 0.0021), Time x Call Type (Three-way RM ANOVA; F (5, 93) = 12.18, *p* < 0.0001), and Time x Sex x Call Type (Three-way RM ANOVA; F (5, 93) = 4.755, *p* = 0.0007) (Fig. 3a). We also note here that a “Monday” effect was observed in both sexes, whereby subjects emitted higher numbers of anticipatory of USVs following 2-day break periods (Fig. 3a). Furthermore, post-hoc Tukey’s demonstrated that males emitted significantly more anticipatory 50 kHz calls relative to females on Mondays (Tukey’s; Day 6: Mean Difference = 18.88, *p* < 0.0001; Day 11: Mean Differences = 17.46, *p* < 0.0001) in addition to emitting significantly more anticipatory 50 kHz calls relative to behavioral session calls on Days 6, 11, and 15 (Tukey’s; Day 6: Mean Difference = 21.56, *p* < 0.0001; Day 11: Mean Difference = 21.47, *p* < 0.0001; Day 15: Mean Difference = 10.10, *p* = 0.0041) (Fig. 3a). Furthermore, there were no sex differences observed in 50 kHz USV emission on Day 1; sex differences in calling patterns only emerged with heroin experience.

We also chose to examine 50 kHz call rates directly before, during, and after heroin infusion experience, as well as nonspecific 50 kHz USV call rates outside of infusion contexts (see Fig. 3a’ for a timeline of call analysis). Call rates were normalized for amount of time within each period of interest to allow for direct comparison. Here, we found that there was a significant effect of call context (Two-Way RM ANOVA; F(1, 25) = 12.43, *p* = 0.0008), sex (Two-Way RM ANOVA; F(1, 24) = 13.63, *p* = 0.0011), as well as a significant interaction between call context and sex (Two-Way RM ANOVA; F(3, 59) = 9.873, *p* < 0.0001). Post-hoc Tukey’s analysis demonstrated significant sex differences in 50 kHz call rates specifically in the time period before heroin infusion (Tukey’s; Mean Difference = 17.16, *p* = 0.0159), as well as directly after heroin infusion (Tukey’s; Mean Difference = 14.26, *p* = 0.0107) (Fig. 3a”). Furthermore, males called at significantly higher rates directly before and after heroin infusion compared to call rates outside of infusion contexts (Tukey’s; Mean Difference = 15.77, *p* = 0.0281; Mean Difference = 11.71, *p* = 0.0293, respectively) (Fig. 3a”). Post-hoc analysis did not demonstrate any significant differences between 50 kHz call rates in females at any of the time points described above. 22 kHz USVs were also examined. Both male and female subjects emit very few 22 kHz USVs throughout acquisition, and no significant differences were observed in 22 kHz emission for any of the above parameters (Three-way RM ANOVA; Time: F (5, 145) = 1.325, *p* = 0.2569; Sex: F (1, 143) = 0.2744, *p* = 0.6012; Call Type: F (1, 29) = 0.2821, *p* = 0.5994; Time x Sex: F (5, 143) = 2.178, *p* = 0.0598; Time x Call Type: F (5, 143) = 1.312, *p* = 0.2624; Sex x Call Type: F (1, 143) = 0.7929, *p* = 0.3747; Time x Sex x Call Type: F (5, 143) = 0.8914, *p* = 0.4887) (Fig. 3b). Call rates were also assessed in the subset of subjects that underwent PR testing; similar patterns to those observed during acquisition were found.

### Correlations between USV profiles and behavioral phenotypes

We examined correlations between 50 kHz ultrasonic vocalizations (USVs) and behavioral phenotypes on Days 1 and 15 of heroin self-administration to evaluate how correlations between phenotypes change with continued heroin experience. Only 50 kHz USV emissions were assessed due to low 22 kHz USV emission during acquisition. In females, on Day 1 we observed a significant positive correlation between anticipatory 50 kHz USV emission and heroin infusions (*R* = 0.560, *p* = 0.019) (Supplemental Fig. 1a and b). On Day 15, this same correlation became negative, but did not reach statistical significance (*R* = −0.253, *p* = 0.345) (Supplemental Fig. 1a). In males on Day 1 there was no relationship between heroin infusion and emission of USVs, but on Day 15 we observed a significant positive correlation between number of heroin infusions and emission of 50 kHz USVs during the behavioral session (*R* = 0.616, *p* = 0.044) (Supplemental Fig. 1a and c). Other correlations (none reaching significance) can be found in Supplemental Fig. 1a. These results suggest that the relationship between 50 kHz USV call rates and heroin self-administration patterns changes over time in a sex-specific manner.

### Extinction

**Fig. 4:**
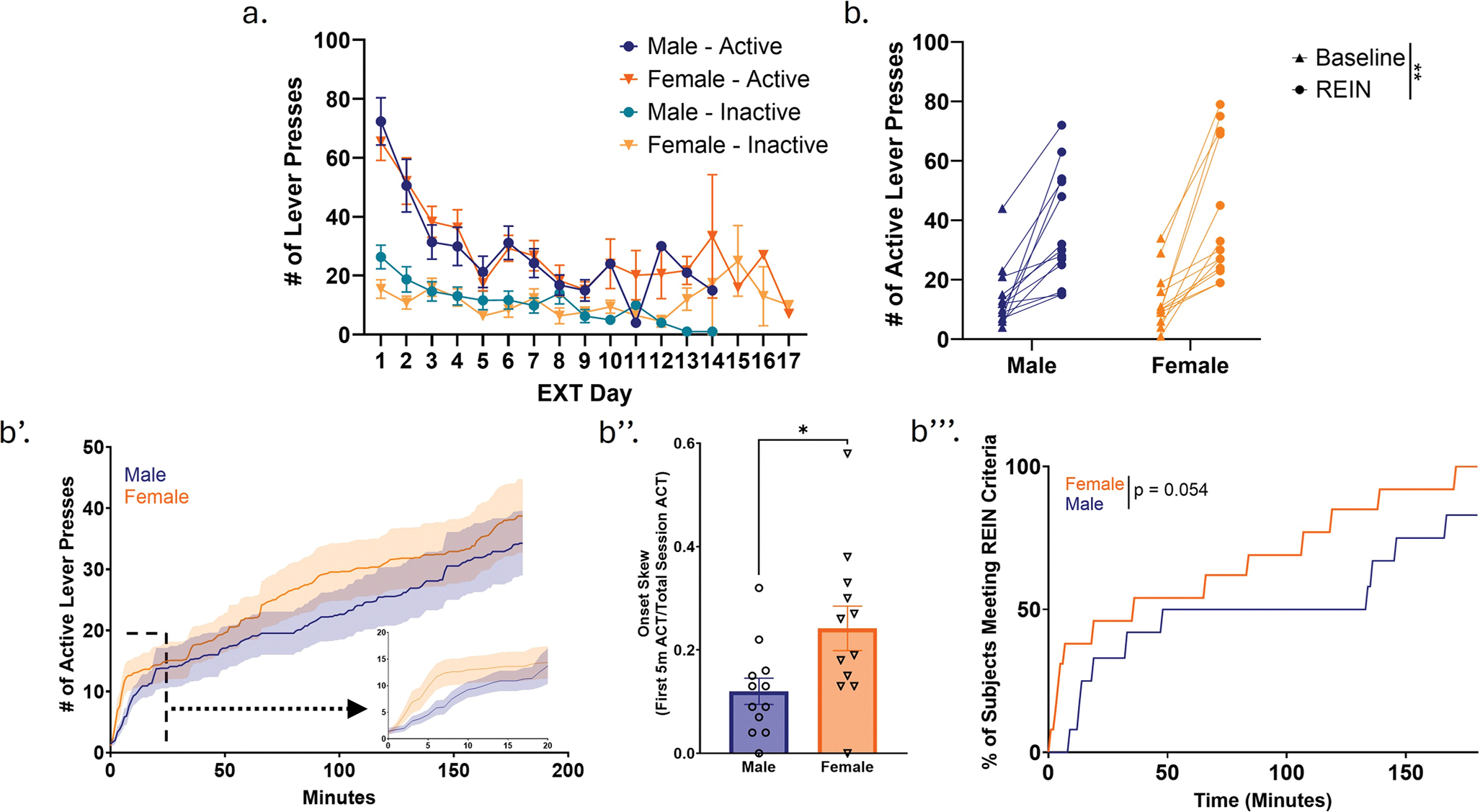
Both males and females achieve successful footshock-stress induced reinstatement of heroin seeking, although through different strategies. a. Extinction lever pressing patterns in males and females. b. Stress-induced reinstatement in males and females; comparison of active lever presses at baseline (final day of extinction) vs. during reinstatement. b’. Microanalysis of active lever pressing patterns during stress-induced reinstatement. Inset indicates difference in lever pressing between males and females during the first 20 min of reinstatement sessions. b”. Onset skew analysis quantifying the frontloading of active lever presses observed in b’. Onset skew was calculated as the number of active lever presses during the first five minutes of the session, expressed as a proportion of total active lever presses across the session. Females exhibited significantly greater onset skew compared to males. One female was excluded as an outlier and is not included in the graph or analysis. b”’. Time to meet reinstatement criteria (minimum of 10 active lever presses and active lever pressing that reached at least 150% of baseline levels). Differences in reinstatement curves assessed via Chi-square test (see text for details). n(♂) = 12, n(♀) = 13. Error bars represent SEMs. * indicates p < 0.05 by unpaired student’s t test. ** indicates main effects in two-way ANOVA (p < 0.01). EXT = Extinction. REIN = reinstatement. ACT = active lever presses

Following six days of home-cage abstinence, subjects were returned to operant chambers to undergo extinction. Extinction continued until extinction criteria were met (see Materials and Methods). Statistical analysis demonstrated no overall sex differences in active lever pressing during extinction (Mann-Whitney U = 116.5, *p* = 0.9298) or time to meet extinction criteria (Mann-Whitney U = 60.50, *p* = 0.4314) (Fig. 4a). Comparison of 50 kHz USVs between the final day of self-administration, the first day of extinction, and the final day of extinction (termed the “baseline session”) demonstrated significant effects of time point (Two-way RM ANOVA; F (3, 53) = 25.86, *p* < 0.0001) and sex (Two-way RM ANOVA; F (1, 26) = 17.14, *p* = 0.0003), as well as a significant interaction between the two factors (Two-way RM ANOVA; F(9, 166) = 7.758, *p* < 0.0001) (Supplemental Fig. 2). Post-hoc Tukey’s demonstrated that significant sex differences were driven by higher emission of anticipatory 50 kHz USVs by males relative to females at all time points (Tukey’s; Last Day of Self-Administration: Mean Difference = 11.26, *p* = 0.0133; Extinction Day 1: Mean Difference = 17.25, *p* = 0.0194; Baseline Session: Mean Difference = 7.708, *p* = 0.0248). There were no significant sex differences in 50 kHz call emission during the behavioral session on the last day of self-administration or the baseline session (Tukey’s; Last Day of Self-Administration: Mean Difference = 1.833, *p* = 0.2020; Baseline Session: Mean Difference = −0.3938, *p* = 0.6246), but males did call significantly more than females during behavioral sessions on the first day of extinction (Tukey’s; Mean Difference = 9.707, *p* = 0.0087). In females, anticipatory 50 kHz call emission did not significantly differ between the last day of self-administration and the first day of extinction (Tukey’s; Mean Difference = −6.700, *p* = 0.0940), but this same comparison was significant in males (Tukey’s; Mean Difference = −16.95, *p* = 0.0088), indicating an increase in anticipatory call rates following abstinence periods. In both males and females, there was no significant difference in anticipatory 50 kHz call rates between the last day of self-administration and the baseline session (Tukey’s; Males: Mean Difference = 3.750, *p* = 0.9378; Females: Mean Difference = −0.9083, *p* > 0.9999). In males, anticipatory 50 kHz call rates on the first day of extinction were significantly higher than anticipatory call rates during the baseline session (Tukey’s; Mean Difference = 16.61, *p* = 0.0453). However, this effect was not observed in females (Tukey’s; Mean Difference = 7.644, *p* = 0.1001). 22 kHz USVs were emitted very infrequently at all time points, and consequently no significant effects were observed in terms of sex (Two-way RM ANOVA; F (1, 26) = 0.0005, *p* = 0.9821), call time point (Two-way RM ANOVA; F (3, 70) = 1.785, *p* = 0.1624), or an interaction between the two parameters (Two-way RM ANOVA; F (5, 128) = 1.186, *p* = 0.3199).

### Stress-induced reinstatement of heroin seeking

Successful stress-induced reinstatement of heroin seeking following the experience of footshock stress was observed in both sexes, as demonstrated by an overall significant increase in active lever pressing from the baseline session to the reinstatement day (Two-way RM ANOVA; F (1, 23) = 45.00, p < 0.0001) (Fig. 4b). This effect occurred to a similar extent in males and females, as we observed no main effects of sex (Two-way RM ANOVA; F (1, 23) = 0.8838, p = 0.3569) or an interaction between sex and reinstatement active lever pressing (Two-way RM ANOVA; F (1, 23) = 0.6898, p = 0.4148) (Fig. 4b). Microanalysis of lever pressing patterns between males and females revealed that both sexes reach similar levels of active lever pressing during reinstatement, but pressing patterns appear to differ during behavioral sessions, with females displaying increased lever pressing during the initial 20 minutes of the session relative to males (Fig. 4b’). To further investigate this observation, we assessed onset skew (again defined as proportion of total active lever presses occurring within the first five minutes of each session) during reinstatement in males and females. Unpaired Student’s *t*-tests revealed a significant sex difference (*t*(22) = 2.439, *p* = 0.0232; Fig. 4b”). One female subject was excluded from the analysis as an outlier. Females exhibited significantly higher onset skew compared to males, with a mean of 0.242 ± 0.043 (Mean ± SEM; expressed as a proportion of total active lever presses), whereas males had a mean of 0.120 ± 0.025. We then assessed the time required to meet reinstatement criteria, defined by two components: (1) a minimum of 10 active lever presses during the reinstatement session, and (2) active lever pressing that reached at least 150% of baseline levels (i.e., a 50% increase relative to baseline). We observed that all females met reinstatement criteria, while 83% of males met this criterion. Furthermore, a Chi-square test between reinstatement curves demonstrated a trend towards a difference between reinstatement curves in males and females (Chi-square = 3.726, *p* = 0.0536) (Fig. 4b”’). As we did not specifically aim to test the effect of circulating gonadal hormones on level of reinstatement observed in females, we are underpowered to reliably assess estrous cycle effects on reinstatement vulnerability.

### USVs during stress-induced reinstatement of heroin seeking

**Fig. 5:**
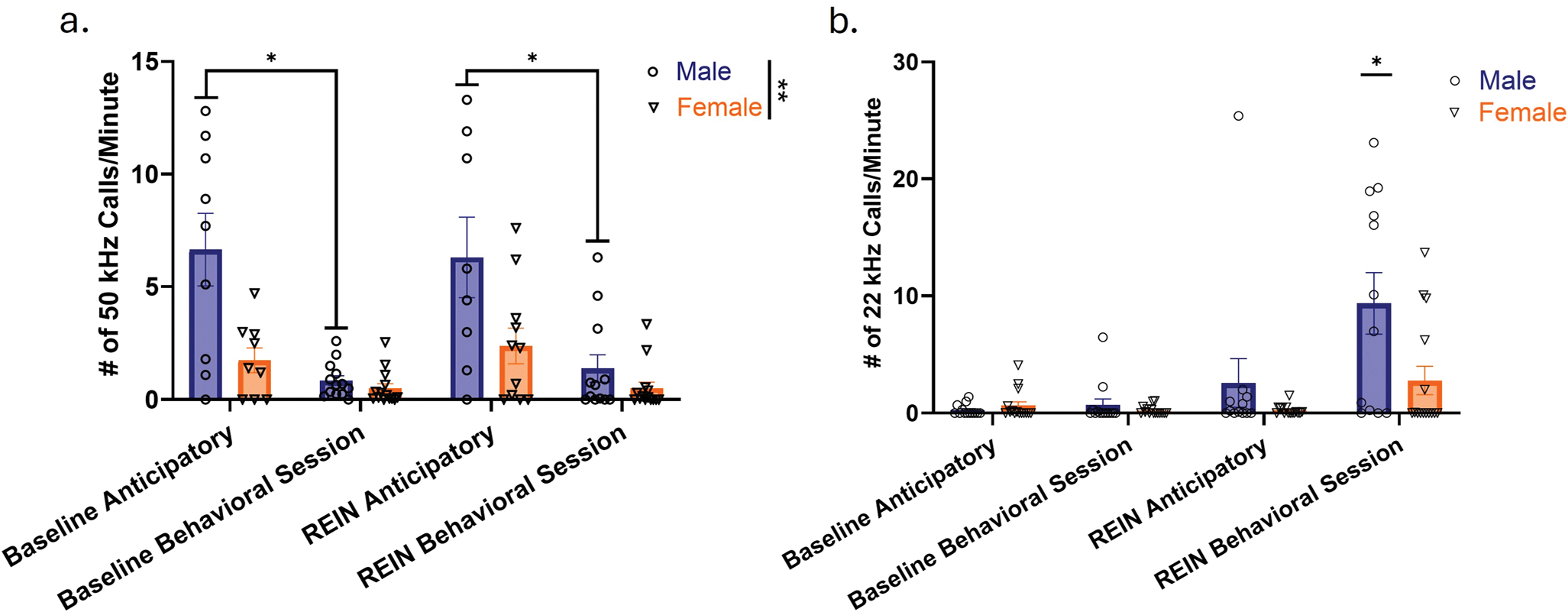
Stress-induced reinstatement behaviors are accompanied by a characteristic pattern of anticipatory 50 kHz USVs, followed by 22 kHz USV emission by males during reinstatement sessions. (a) 50 kHz USV emission patterns in baseline and reinstatement sessions. (b) 22 kHz USV emission patterns in baseline and reinstatement sessions. REIN anticipatory USVs were measured in an interim period between footshock and the start of behavioral sessions. n(♂) = 13, n(♀) = 15. Error bars represent SEMs. * indicates p < 0.05 by post-hoc Tukey’s Multiple Comparisons (Panel a) or Dunn’s (Panel b; Male REIN Behavioral Session compared to male and female 22 kHz USVs at all other time points, see text for details of comparisons). ** indicates main effects in two-way ANOVA (p < 0.01). REIN = reinstatement

50 and 22 kHz USVs were recorded during an interim period between footshock experience to capture anticipatory USVs, as well as during reinstatement behavioral sessions. USVs were compared to USV emission patterns during baseline sessions. Analysis of 50 kHz USVs demonstrated a significant effect of session (Two-way RM ANOVA; F (2, 41) = 21.43, *p* < 0.0001) and sex (Two-way RM ANOVA; F (1, 26) = 12.75, *p* = 0.0014), as well as a significant interaction between the two factors (Two-way RM ANOVA; F (3, 56) = 6.409, *p* = 0.0008) (Fig. 5a). Again, previous post-hoc Tukey’s analysis demonstrated that males emitted significantly more anticipatory 50 kHz calls than females during the baseline session, replicating sex differences observed in previous paradigm analysis points (Tukey’s; Mean Difference = 7.708, *p* = 0.0248). A trend towards a significant sex difference in anticipatory calls on the reinstatement day was observed, but did not reach statistical significance (Tukey’s; Mean Difference = 3.918, *p* = 0.0737). No sex differences were observed during behavioral sessions on the baseline or reinstatement day (Tukey’s; Baseline Session: Mean Difference = 0.2553, *p* = 0.3426; Reinstatement Session: Mean Difference = 0.8720, *p* = 0.2125). For males, significantly higher anticipatory 50 kHz calls were emitted relative to calls during the behavioral session on both baseline and reinstatement sessions (Tukey’s; Baseline Session: Mean Difference = 6.117, *p* = 0.0188; Reinstatement Session: Mean Difference = 5.588, *p* = 0.0333). In females, there were no significant differences between anticipatory and behavioral session call rates at either time point (Tukey’s; Baseline Session: Mean Difference = 1.583, *p* = 0.0646; Reinstatement Session: Mean Difference = 1.814, *p* = 0.1064). Anticipatory 50 kHz call rates between the baseline and reinstatement session did not significantly differ for either sex (Tukey’s; Males: Mean Difference = 0.1429, *p* = 0.9996; Females: Mean Difference = −0.03750, *p* = 0.9999). For 22 kHz calls, analysis demonstrated that calls significantly differed across session and sex (Kruskal-Wallis statistic = 28.43, *p* = 0.0002), with a post-hoc Dunn’s demonstrating that differences were specifically driven by a significant increase in 22 kHz call emission by males during reinstatement behavioral sessions (Fig. 5b; details of Dunn’s comparisons can be found in Supplemental Table 1). In a subset of animals, USVs were recorded prior to and during footshock. However, all of these animals were low callers throughout acquisition, extinction, and reinstatement. Therefore, their data is not represented in this analysis. USV emission at other timepoints is included in an expanded graph (Supplemental Fig. 2).

### Correlations between behavioral and USV phenotypes and level of reinstatement

We assessed correlations between level of active lever pressing and various behavioral and USV profiles to try and determine phenotypes associated with reinstatement vulnerability. We observed a significant positive correlation between active lever pressing on Day 1 of extinction and level of active lever pressing during reinstatement in females (*R* = 0.760, *p* = 0.0003) (Supplemental Fig. 3a and b). No other significant relationships were observed (Supplemental Fig. 3a).

## Discussion

This study is among the few to investigate sex differences in heroin self-administration and footshock stress-induced reinstatement behaviors, as well as related affective states, throughout drug use cycles. By employing a long-intermittent access model and incorporating ethologically relevant measures such as ultrasonic vocalizations (USVs), the study provides valuable translational insights into these behaviors within a preclinical context. This study demonstrates marked sex differences in heroin-related behaviors and affective states, ultimately indicating sex-specific vulnerabilities to the development of heroin use disorders.

### Females show significantly higher intake and escalation of heroin self-administration compared to males

We observed that females lever pressed, and consequently consumed significantly more heroin relative to males. Previous literature regarding sex differences in heroin/opioid self-administration has produced mixed results—generally, both preclinical and clinical findings report faster acquisition of opioid self-administration, including heroin, in females relative to males (Lynch & Carroll, 1999; Carroll et al., 2002; Cicero et al., 2003; Becker & Hu, 2008; Becker et al., 2012; McHugh et al., 2018; Zanni et al., 2020; George et al., 2021; Knouse & Briand, 2021), but this effect is not consistent across studies (Stewart et al., 1996; Venniro et al., 2017; George et al., 2021; Bossert et al., 2022; D’Ottavio et al., 2023). It is important to highlight that these studies differ substantially from ours in several key aspects, including the rat strain used, the implementation of our specific long-intermittent access schedule, and the dosing regimen, which involved initial training at a higher heroin dose (0.1 mg/kg/INF) before transitioning to a lower dose (0.05 mg/kg/INF). We believe that our specific choice of access schedule and dosages contributes to the observation of sex differences. We observe the emergence of these sex differences over the course of Weeks 2 and 3 of self-administration (at which time the lower dose of heroin is utilized), during which time male responding plateaus, while female responding continues to increase until reaching a plateau at a higher level. These observed sex differences may result from differences in metabolism of heroin, or from differential neural responses/adaptations to chronic heroin exposure and consumption (for review see (Becker & Chartoff, 2019; Becker & Mazure, 2019; Pisanu et al., 2019)).

Microanalysis of behavioral patterns on Day 11 of acquisition revealed that both males and females exhibited frontloading of active lever pressing, particularly during Session 1, with over one-third of total presses occurring within the first 5 min—a particularly striking finding given that this window comprises only about 4% of total session time. Frontloading was also observed during Sessions 2 and 3, although to a lesser extent. As this analysis period took place on Mondays following two-day breaks, these patterns may suggest a “mini-incubation” effect that manifests in heightened heroin-seeking behavior and onset skew upon re-entry into the drug-paired context. However, only females showed a significant effect of session in post-hoc comparisons, with higher onset skew in Session 1 relative to Sessions 2 and 3. This suggests a more profound incubation-like effect in females during initial drug access. However, no overall sex differences or sex by session interactions in onset skew behaviors were observed; this finding is consistent with a recent report showing similar frontloading patterns between sexes during intermittent access heroin self-administration (D’Ottavio et al., 2023). Despite pronounced frontloading in active lever pressing, heroin infusion patterns during individual behavioral sessions followed a consistent linear trajectory across all three sessions. Both males and females exhibited steady heroin consumption at similar rates across sessions, although females demonstrated significantly higher rates than males, consistent with the observed sex differences in active lever pressing and infusions. Studies examining behavioral micropatterns in the context of heroin consumption are limited, this finding is somewhat contrary to a previous study, in which a “frontloading” of both infusions and active lever presses was observed (D’Ottavio et al., 2023). However, while this study also utilized an intermittent access schedule, it was not the 3 × 2 h design that we implemented; these differential access periods may account for differences in patterns of lever pressing/consumption during individual sessions.

While we observed sex differences in lever pressing and heroin consumption, importantly, we did not observe any correlation between estrous cyclicity and heroin self-administration patterns in females. Work regarding the influence of fluctuating gonadal hormones on opioid-related behaviors is somewhat limited, and literature in the field demonstrates inconsistent results/impacts of gonadal hormones on behavior (for review see (Knouse & Briand, 2021). With this being said, our results are consistent with some other work in the field demonstrating no effect of estrous cyclicity on opioid-related behavior (Stewart et al., 1996; Mayberry et al., 2022), thus contributing to the idea that the observed sex differences in heroin self-administration behaviors are driven by organizational as opposed to activational sex differences.

### Sex differences in USV emission during heroin self-administration support positive reinforcement-driven responding in males

Marked sex differences in USV emission, reflective of affect, were also observed during acquisition of heroin self-administration. Males emitted significantly more “positive” 50 kHz USVs during acquisition relative to females, a finding that is especially notable given the absence of sex differences in 50 kHz USV emission on the first day of self-administration, prior to any heroin experience. In addition, post-hoc analyses revealed that males emitted significantly higher anticipatory 50 kHz USVs relative to behavioral sessions calls on Mondays following two-day breaks, suggesting elevated positive affective states in anticipation of heroin availability within the drug-paired operant chamber. While females exhibited a similar trend, this difference did not reach statistical significance. Moreover, males emitted significantly more anticipatory 50 kHz USVs on Mondays relative to females, further highlighting a sex-specific affective response. This heightened anticipatory drive in males persisted into Week 3, as evidenced by significantly greater anticipatory USVs relative to behavioral session USVs on Day 15, a pattern again not observed in females. Collectively, these findings point to a sex-specific elevation in positive affective motivation for heroin consumption. This observation mirrors a commonly reported clinical phenomenon, where men are more likely to report positive reinforcement-driven drug use, while women tend to report negative reinforcement-driven drug use (Becker & Hu, 2008; Becker & Chartoff, 2019; Cornish & Prasad, 2021). Given that we did not observe sex differences in 50 kHz USV emission on Day 1 of acquisition, we believe this finding is being effectively recapitulated in a preclinical context. Furthermore, we observed a significant positive association between behavioral 50 kHz USV emission in males on the final day of acquisition and number of heroin infusions (an association that was not present on Day 1), which supports the idea of context-driven positive affect surrounding drug consumption in males. Additionally, we observed that males emitted significantly more 50 kHz USVs around “infusion contexts” (i.e., before and after heroin infusions) compared to non-specific calling during behavioral sessions. This suggests that the call emission may be linked to both the anticipation and motivation to consume heroin, as well as the positive hedonic sensations following heroin infusion.

Given that both females and males emitted 22 kHz USVs infrequently during acquisition, it is difficult to assess the role of negative reinforcement-driven drug seeking during this time period; it is possible that negative reinforcement is not playing a large role in drug seeking and consumption during this phase. With this being said, we did observe a significant positive correlation between anticipatory 50 kHz USV emission and heroin consumption on Day 1 in females; this correlation became negative (but did not reach significance) by the final day of self-administration; while this observation does not fully support the idea of negative-reinforcement driven responding in these females, it does differ from male USV patterns in a manner that demonstrates some differences in affective responding to drug associated contexts and cues.

Male rats have been shown to emit more 50 kHz USVs than females in the context of other rewarding behaviors, such as rough play and prior to mating initiation (Barfield & Thomas, 1986; Snoeren & Ågmo, 2014; Willadsen et al., 2014; Kisko et al., 2015). However, to our knowledge, sex differences in USV emission during drug self-administration have not been thoroughly investigated. In our study, we examined 50 kHz USV emission in a general sense, though we acknowledge that there are various subtypes of 50 kHz USVs, with some studies identifying as many as 14 distinct subtypes, all associated with specific communicative relevance (Wöhr et al., 2008; Burgdorf J & Moskal JR, 2010; Wright et al., 2010; Burgdorf et al., 2011; Simola & Costa, 2018). We observed a broad range of 50 kHz USV emission subtypes throughout the recording paradigms; however, we did not specifically categorize or quantify the emission of these subtypes. Future work could focus more on categorization of these subtypes (as well as sex differences in emission profiles) in the context of drug-motivated behavior to identify specific emission profiles associated with certain behavioral phenotypes of vulnerability/resilience to drug consumption.

### Both males and females reach similar levels of stress-induced reinstatement of heroin seeking, although achieved through different strategies.

Footshock stress-induced reinstatement paradigms were originally established in heroin contexts, but have been found to generalize to other drugs, including cocaine, nicotine, and ethanol (for review see (Mantsch et al., 2016). The footshock stress-induced reinstatement paradigm that we have implemented does not precisely align with other footshock studies (in terms of drug dosage, schedule of access, level of footshock, etc.), but does expand work within the field. When examining number of active lever presses, we did observe significant stress-induced reinstatement in males and females, but no sex differences in level of reinstatement. Studies examining sex differences in vulnerability to footshock stress-induced reinstatement of opioid seeking are extremely limited. To our knowledge, only one study has assessed this effect in the context of short access to oral oxycodone; this study reported no sex differences but employed a different drug/access schedule (Fulenwider et al., 2020). Two studies have assessed sex differences in yohimbine stress-induced reinstatement of opioid seeking, yielding conflicting results: one reported no sex differences (Malone et al., 2021), while the other found higher yohimbine-induced responding in females (Smethells et al., 2020).

Our microanalyses of lever pressing behavior demonstrated frontloading of active lever pressing in females during reinstatement behavioral sessions; with females rapidly escalating active lever pressing immediately when behavioral sessions were initiated. This observation was confirmed with onset skew analysis, with females displaying significantly higher onset skew relative to males during the reinstatement behavioral session. This effect is reminiscent of a recent study demonstrating greater frontloading behavior in footshock-exposed females during cocaine self-administration (Gaulden et al., 2025). As frontloading is commonly thought to be indicative of elevated motivation to consume a drug, this pattern may reflect differential stress vulnerability between males and females, with stress potentially driving a stronger drug-seeking motivation in females following footshock. We interpret this effect as reflecting differences in negative reinforcement-driven drug taking rather than differences in relative pain sensitivity, as prior work from our laboratory has not found any relationship between pain thresholds and heroin-taking phenotypes (Li et al., 2025).

We also observed a significant positive correlation between active lever pressing on Day 1 of extinction and active lever responding during reinstatement in females only. This finding aligns with prior work in the field: in rodent models of opioid self-administration (including heroin, morphine, and oxycodone), animals exhibiting greater drug-seeking following forced abstinence typically display more robust reinstatement effects, although most of these studies have employed a cue-induced reinstatement model (For review see (Reiner et al. 2019; Venniro et al. 2021; Bergeria et al. 2024; Chow et al. 2025)). Our findings suggest that this relationship may also extend to stress-induced reinstatement and may do so in a sex-specific manner, further supporting evidence for sex-dependent associations between stress and drug-seeking behavior.

In addition to sex differences in lever pressing patterns, we observed distinct patterns of USV emission, with males specifically emitting significantly higher numbers of 22 kHz USVs during reinstatement behavioral sessions relative to other time points. Furthermore, both sexes (but particularly males) continue to emit 50 kHz USVs throughout all “anticipatory” periods, even those following footshock experience. The sex differences we observed in the context of 22 kHz USVs appears to be consistent with previous data in the field demonstrating lower 22 kHz USV emission in females in response to experimental stressors (including footshock), especially in Sprague-Dawley rats (Graham et al., 2009; Inagaki, 2018). It is possible that emission of these 22 kHz USVs during behavioral sessions may be reflective of negative affective responses to stress or a kind of “frustration” effect (i.e. subjects attempt to seek drug to relieve negative affective states but do not receive any). This kind of frustration 22 kHz USV emission has been reported in males in the context of copulatory behaviors, where male rats will emit 22 kHz USVs if females are non-receptive or if mating is unsuccessful (Barfield et al., 1979). Although these studies do not directly align with our work, they suggest that frustration effects may generalize to other types of reward, which warrants further examination. Overall, we found that USVs during both acquisition and reinstatement periods do not always strongly correlate with behavioral profiles such as drug consumption and relapse levels in an expected manner. This makes it challenging to assess the relationship between affective state and drug-taking behaviors using USVs. Consequently, the interpretation of USVs is not straightforward, suggesting that a deeper understanding of their context—considering history and environmental experience—may be necessary.

### Limitations and future directions

We acknowledge that female rats were freely cycling, so testing occurred during high or low estrogen stages. There is mixed evidence that gonadal hormones may affect heroin consumption and seeking behaviors (Knouse & Briand, 2021; Nicolas et al., 2022); we did not observe any association between estrous cycle stage and behavioral output in our studies. Additionally, since we did not emphasize equal inclusion of estrous cycle phases, we are underpowered to properly assess estrous cycle influence during stress-induced reinstatement.

Additionally, we wish to highlight the significance of our choice of long-intermittent access self-administration schedules, coupled with the shift from “high” to “low” heroin infusion doses. We believe that this schedule and dose adjustment may help to evoke sex differences, which are not always observed in other self-administration paradigms that use different schedules or doses of heroin (George et al., 2021; Knouse & Briand, 2021; Nicolas et al., 2022; Towers et al., 2022; D’Ottavio et al., 2023). Furthermore, while there is mixed evidence suggesting potential sex differences in footshock sensitivity (Weinstock et al., 1998; Manzano-Nieves et al., 2018; Presto et al., 2021; Olivera-Pasilio & Dabrowska, 2023), we did not explore this in our current paradigm, as both males and females were exposed to the same intensity of footshock. However, given the absence of sex-specific behavioral differences in response to footshock, we do not believe that potential sex differences in sensitivity influenced our behavioral outcomes.

Finally, we recognize that this set of experiments focuses solely on behavioral analyses, without identification of contributions of underlying neurobiological systems. Previous studies within our laboratory have established a role for the dorsal raphe nucleus (DRN) serotonin (5-HT) system in modulating basal and compulsive heroin self-administration in male rats, highlighting the role of this system in mediating both rewarding and aversive consequences (Li et al., 2025). We plan to build on these findings to determine whether differences in DRN 5-HT transmission contribute to the sex differences in heroin-related behaviors and affective states observed here.

## Conclusions

In summary, the current study demonstrated marked sex differences in heroin self-administration patterns, with females displaying enhanced heroin self-administration relative to males. Furthermore, we observed footshock stress-induced reinstatement of heroin seeking in both sexes, although achieved through different strategies, thus implying potential sex-specific encoding of stressors and reward value. We also observed distinct sex differences in USV emission profiles, although these profiles did not always clearly correlate with heroin consumption/reinstatement patterns. These findings indicate sex-specific vulnerabilities to the development of heroin use disorders, in addition to outlining affective states that may contribute to and result from heroin consumption and seeking.

## Supplementary Material

Supplementary Material

**Supplementary Information** The online version contains supplementary material available at https://doi.org/10.1007/s00213-025-06920-w.

## Data Availability

Data available on request from the authors.
