# Supplementary material for: Marked sex differences are observed in heroin acquisition and affective states in rats but converge to similar levels of footshock stress-induced reinstatement
Source: Psychopharmacology (Berl). 2025 Nov 3;243(7):1711–26. doi: 10.1007/s00213-025-06920-w (PMC12951745; doi:10.1007/s00213-025-06920-w)
Supplement: Supplementary file 1 [file 213_2025_6920_MOESM1_ESM.docx]

**Supplemental Methods.**

**S.1 | *Self-Administration.***

Self-administration was conducted in the dark phase in operant conditioning chambers enclosed in sound-attenuating, ventilated environmental cubicles (Med Associates, Inc., St. Albans, VT). All operant chambers were equipped with ultrasonic USB microphones (Ultramic UM200K, Dodotronic) positioned above the ceiling of chambers in order to allow for capture of USV emissions. The start of behavioral sessions was indicated by house lights turning on and active and inactive levers extending into the chamber. When the active lever was pressed, presentation of a 2 second light/tone (75 dB) cue ensued, followed by triggering a syringe pump (MedAssociates, Inc. St. Albans, VT) to allow for delivery of heroin solutions via a stainless-steel swivel (Instech Laboratories, Inc., Plymouth Meeting, PA) and polyethylene tubing attached to catheter portals on each subject’s back. Pump time was adjusted for each subject according to their weight in order to achieve proper dosage via the following formulas: (0.1 mg/kg/infusion: weight * 0.0282; 0.05 mg/kg/infusion: weight* 0.0141). Following drug infusion, a 20 second time-out period took place, during which house lights were turned off; subjects were still able to press active levers, but lever pressing would not result in light/tone presentation or drug infusion. Inactive lever pressing did not result in any consequences. All behavioral parameters, including number of infusions, number of active lever presses, and number of inactive lever presses, were recorded in MedAssociates Software (Med PC IV). During the fixed ratio 1 (FR1) schedule, a drug infusion follows every lever press except presses during time-out periods. Progressive ratio (PR) testing was performed in a subset of subjects shown in the Supplemental Materials. During PR schedules, rats need to perform higher numbers of active lever presses to receive the next infusion of heroin. The schedule was as follows: 1, 2, 4, 6, 9, 12, 15, 20, 25, 32, 40, 50, 62, 77, 95, 118 etc. Sessions ended if subjects did not meet breakpoint (BP) criteria within 30 minutes or if the full 6-hour session time was met. BP was defined as the maximum number of presses resulting in the final infusion of heroin. All USV emissions were recorded in a separate computer equipped with RavenPro recording software (Cornell Lab or Ornithology, Bioacoustics Research Program); USV signals were recorded at 192-kHz, and later digitized, amplified, and written to “.wav” files after application of a fast Fourier transform.

**S.2 | *Normalization of Call Rates.***

Infusions were examined in DeepSqueak, as pump noises were picked up by microphones and thus were able to be visualized. The number of 50 kHz calls 5 seconds before, during, and 5 seconds after this pump time was recorded for each subject. Non-specific calling was considered to be any 50 kHz calls outside of these contexts and was determined from these above parameters. Total amount of time before infusions (5s* number of infusions), total amount of time after infusions (5s* number of infusions), and total time during infusions (pump time*number of infusions) was calculated, and subtracted from total amount of time (20 min). Call rates for each period were then calculated (i.e. total calls before infusion/total time before infusion; total calls during infusion/total time during infusion; total calls after infusion/total time after infusion; (total calls – calls before, after, and during infusion)/total time after subtracting.

**S.3 | *Estrous Cycle Tracking.***

Female rats were briefly restrained, and the vaginal canal was briefly flushed with sterile saline by inserting a pipette tip into the canal, slowly releasing saline, and then drawing it back up into the pipette tip. Care was taken to not stimulate the cervix so as to not induce pseudopregnancy. Once flushed, the sterile saline solution containing suspended cells was placed on a glass microscope slide (VWR Double Frosted Microscope Slides; VWR International LLC, Radnor, PA, USA), and then air dried for at least 48 hours prior to imaging under a microscope. Cells were visualized at 20x or 40x, and cell shape and distribution of cell type were used to determine estrous cycle phase, following standard procedures. Briefly, estrous was identified by a large proportion of cornified epithelial cells, proestrus was identified by mostly nucleated epithelial cells, with a small proportion of cornified epithelial cells, metestrus was identified by a relatively even proportion of cornified epithelial cells, nucleated epithelial cells, and leukocytes, and diestrus was identified by mostly leukocytes. If subjects appeared to be in pseudopregnancy (marked by an extended period of time in which cytology appeared to resemble diestrus), then data collected during this period of time was not included in subsequent analyses. We did observe estrous cycle disruption/unpredictable cycling in a subset of subjects; these subjects were not included in estrous cycle analysis (4 of 17 subjects not included due to inconsistent cycling). Furthermore, to control for potential stress associated with lavage experience, we performed “pseudo-lavage” in males, also implementing brief restraint. Lavage was performed following the final session of each day (~16:00).

**Supplemental Figure 1. *Correlations between behavioral and USV phenotypes.*** a. Pearson’s R and p values for all correlations performed. b. Scatterplot demonstrating significant relationship between anticipatory 50 kHz USV emission and number of heroin infusions in females on Day 1 of self-administration. c. Scatterplot demonstrating significant relationship between behavioral 50 kHz USV emission and number of heroin infusions in males on Day 15 of self-administration. n(♂) =14, n(♀) = 17. * indicates correlations with p < 0.05. ACT = active lever presses. INF = infusion. ANT = anticipatory. BEH = behavioral session.

**Supplemental Figure 2. *Expanded USV emission profiles during extinction and reinstatement.*** a. 50 kHz USVs. b. 22 kHz USVs. Analysis during the “During Shock Anticipatory” and “During Shock Behavioral Session” was only conducted in a subset of animals. For these timepoints, n(♂) =3, n(♀) = 6. For all other timepoints, n(♂) =13, n(♀) = 16. Error bars represent SEMs. REIN = reinstatement.

**Supplemental Table 1. *Significant post-hoc comparisons of 22 kHz calls during reinstatement.*** Data was assessed using Kruskal-Wallis tests, followed by post-hoc Dunn’s comparisons. n(♂) = 13, n(♀) = 15. REIN = reinstatement.

**Supplemental Figure 3. *Correlations between level of reinstatement and USV phenotypes.*** a. Pearson’s R and p values for all correlations performed. b. Scatterplot demonstrating significant relationship between active lever pressing on Day 1 of extinction and reinstatement active lever pressing in females. n(♂) =12, n(♀) = 13. ** indicates correlations with p < 0.01. ACT = active lever presses. INF = infusion. ANT = anticipatory. BEH = behavioral session. USV = ultrasonic vocalization. EXT = extinction. REIN = reinstatement. AVG = average. ACT = active lever presses.
